# Xanthine dehydrogenase downregulation promotes TGFβ signaling and cancer stem cell-related gene expression in hepatocellular carcinoma

**DOI:** 10.1038/oncsis.2017.81

**Published:** 2017-09-25

**Authors:** G-L Chen, T Ye, H-L Chen, Z-Y Zhao, W-Q Tang, L-S Wang, J-L Xia

**Affiliations:** 1 Department of Oncology, Minhang Hospital, Fudan University, Shanghai, China; 2 Department of Medicine 3, Institute for Clinical Immunology, Friedrich-Alexander-University Erlangen-Nuremberg (FAU) and Universitätsklinikum Erlangen, Erlangen, Germany; 3 Division of Translational Medicine, Minhang Hospital, Fudan University, Shanghai, China; 4 Liver Cancer Institute, Zhongshan Hospital, Fudan University, Shanghai, China

## Abstract

Xanthine dehydrogenase (XDH), a rate-limiting enzyme involved in purine metabolism, has an essential role in inflammatory cascades. Researchers have known for decades that XDH activity is decreased in some cancers, including hepatocellular carcinoma (HCC). However, the role of XDH in cancer pathogenesis has not been fully explored. In this study, we showed that low XDH mRNA levels were correlated with higher tumor stages and poorer prognoses in patients with HCC. Knocking down or inhibiting XDH promoted migration and invasion but not proliferation of HCC cells. The abovementioned phenotypic changes are dependent on increases in epithelial-mesenchymal transition marker gene expression and transforming growth factor-β-Smad2/3 signaling activity in HCC. XDH overexpression suppressed HCC cell invasion *in vitro* and *in vivo*. In addition, the expression and activity of XDH were associated with the expression of CSC-related genes, such as CD44 or CD133, in HCC cells. These data suggest that downregulated XDH expression may be a useful clinical indicator and contribute to the development and progression of HCC.

## Introduction

Hepatocellular carcinoma (HCC), an inflammation-associated malignancy,^1, 2, 3^ is one of the most common cancers worldwide. However, the molecular mechanisms underlying HCC development and progression, including metastasis,^4, 5^ in affected patients have not been fully elucidated. Xanthine dehydrogenase (XDH), a rate-limiting enzyme involved in purine metabolism,^6, 7, 8, 9^ also functions as a key regulator of inflammatory cascades.^8, 9, 10^ XDH activation can produce abundant reactive oxygen or nitrogen species, which may induce DNA damage and carcinogenesis^8, 11, 12, 13^ and promote metastasis.^14^ XDH-derived oxidative stress or uric acid regulates multiple intracellular signals,^8^ such as nuclear factor-kappaB,^15, 16^ hypoxia-inducible factor-α and peroxisome proliferator-activated receptor gamma.^17^ XDH activity is upregulated in the liver during postnatal growth and in the breast during pregnancy and lactation, suggesting it may be a marker of differentiation for liver and mammary epithelial cells.^8^ In contrast to high levels in non-cancerous livers,^18, 19^ decreased XDH activity is believed to confer hepatic cancer cells with selective advantages that are independent of growth rates and degrees of neoplastic histological differentiation.^18, 19^ Indeed, significant decreases in XDH activity levels have been reported to be useful predictors of poor patient prognoses in cancers,^8^ including breast cancer,^20^ gastric cancer,^21^ ovarian cancer,^22^ non-small cell lung cancer^23^ and colorectal cancer.^24^ These poor patient prognoses are believed to involve increased COX-2 (cyclooxygenase-2)^20^ and matrix metalloprotease (MMP)-1/-3 expression.^8, 25^ Despite these intriguing findings, how decreases in XDH activity or expression contribute to the development and progression of cancers, including HCC, remains poorly understood.

Interestingly, XDH inhibition promotes skin wound healing in healthy individuals^26^ and diabetic patients.^27^ Knocking out the XDH gene in mice increased kidney tissue fibrosis and upregulated transforming growth factor-β (TGFβ) and epithelial-mesenchymal transition (EMT) gene expression levels.^28^ The results of these studies suggested that XDH loss may be linked to TGFβ signaling pathway activity.^25, 29^ Indeed, the TGFβ signaling pathway^1, 2, 30^ can increase COX-2 and MMP expression^31, 32, 33, 34, 35^ and promote HCC invasion and progression by inducing EMT and cancer stem cell (CSC) expression.^1, 25, 36, 37^ Moreover, therapies targeting TGFβ signaling appear to be promising in the treatment of HCC.^2^ However, whether XDH deficiency induces TGFβ pathway activity, thereby promoting EMT or CSC marker gene expression in HCC, is unknown.

In the present study, we reported that low XDH expression levels are an unfavorable clinical indicator in patients with HCC. Knocking down or inhibiting XDH resulted in TGFβ signaling pathway-dependent cell migration and invasion caused by EMT-related gene upregulation in HCC cell lines. We also found that the expression levels of CSC-related genes can be altered by the interruption of XDH expression in HCC. These data may improve our understanding of the role of XDH in the development and progression of HCC, as well as other cancers with low XDH expression or activity levels.

## Results

### Decreased *XDH* mRNA expression is associated with aggressive HCC phenotypes

**Figure 1:**
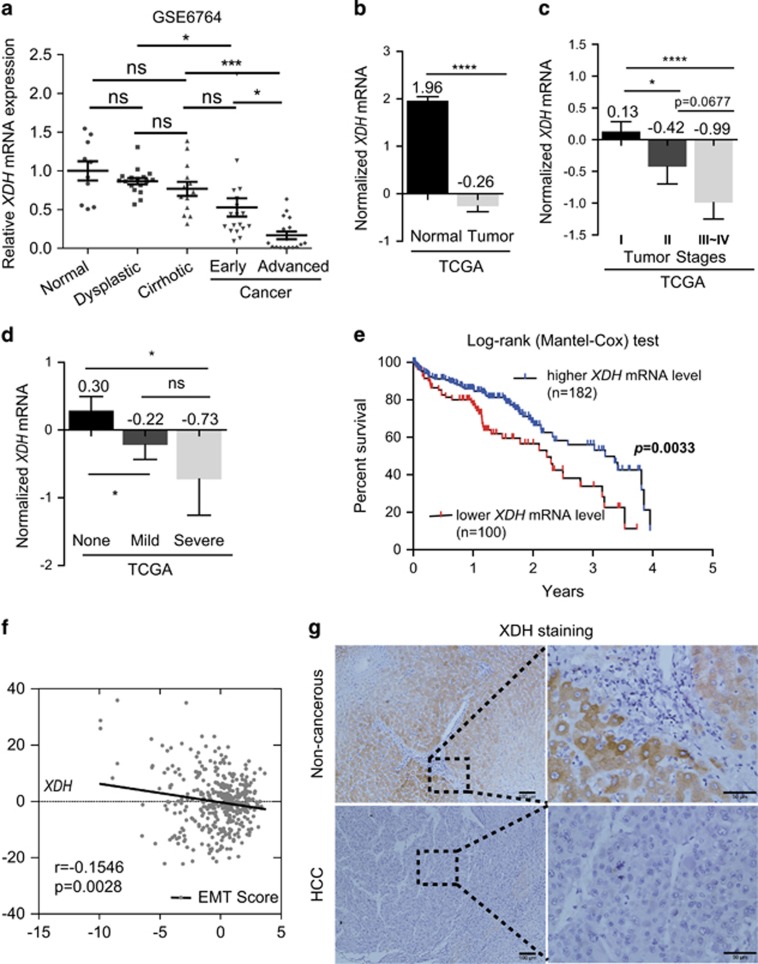
Decreased *XDH* mRNA expression levels predict poor prognosis in patients with HCC. (**a**) Analysis of human *XDH* mRNA levels in normal quiescent (*n*=10), dysplastic (*n*=17), cirrhotic (*n*=13), early (*n*=18) or advanced HCC (*n*=17) livers (clinical data set GSE6764;^ref. 38^). The horizontal lines indicate the mean±s.e.m. *P*-values were calculated by one-way ANOVA (Tukey's multiple comparison test). (**b**) Analysis of *XDH* mRNA levels in normal livers (*n*=50) and cancer livers (*n*=373) from TCGA LIHC patient samples (*n*=423). The horizontal lines indicate the mean±s.e.m. *P*-values were calculated by nonparametric Mann–Whitney *U*-tests. (**c**) XDH transcript levels in TCGA LIHC patient samples comprising tumors of different stages. *P*-values were calculated by unpaired *t*-tests. (**d**) *XDH* mRNA levels in TCGA LIHC patient samples with active adjacent hepatic tissue inflammation of different types and severities. *P*-values were calculated by nonparametric Mann–Whitney *U*-tests. (**e**) Kaplan–Meier survival plots of HCC patients stratified by *XDH* mRNA expression abundance. Log-rank (Mantel–Cox) test. (**f**) Analysis of the correlation between EMT scores and *XDH* mRNA expression levels in TCGA LIHC data set. Pearson’s coefficient analyses were performed to assess statistical significance. (**g**) Representative immunohistochemical staining for XDH in paraffin-embedded liver samples from patients with primary HCC (*n*=9). Scale bar, 50 μm. TCGA, the Cancer Genome Atlas; LIHC, liver hepatocellular carcinoma; XDH, xanthine dehydrogenase; HCC, hepatocellular carcinoma; mRNA, messenger RNA. EMT, epithelial-to mesenchymal transition. ns, not significant, *P<0.05, **P<0.01, ***P<0.001, ****P<0.0001.

To determine whether XDH can serve as a clinical indicator in patients with HCC, we analyzed *XDH* mRNA expression abundance in HCC patient samples deposited in public databases, including the Cancer Genome Atlas (TCGA) and the Gene Expression Omnibus. As shown in Figures 1a and b, lower *XDH* mRNA levels were observed in patients with liver cancer, particularly patients with advanced liver cancer (GSE6764),^38^ than those in healthy controls. XDH transcript levels were negatively correlated with tumor stages in HCC (Figure 1c), suggesting that XDH may be a useful clinical indicator in patients with HCC. Lower *XDH* mRNA expression levels were associated with more active hepatic inflammation in adjacent tissues (Figure 1d), a finding generally observed in patients with HCC with shorter disease-free survival.^10^ HCC patients with lower *XDH* mRNA levels had a poorer prognosis than that of patients with higher *XDH* mRNA levels (Figure 1e). Furthermore, *XDH* mRNA levels were inversely correlated with EMT scores (Figure 1f), which are useful indices for assessing EMT as proposed by Salt *et al.*,^39^ as well as with all detectable *MMP* mRNA levels, in TCGA LIHC patient samples (Supplementary Table 3). Indeed, immunohistochemical staining for XDH in liver samples from a cohort of patients with HCC (*n*=9) showed lower protein levels than adjacent non-cancerous tissue (Figure 1g). Taken together, these findings indicate that loss of XDH expression may be a feature of aggressive HCC.

### XDH downregulation promotes cell migration, invasion and EMT marker gene expression in HCC

**Figure 2:**
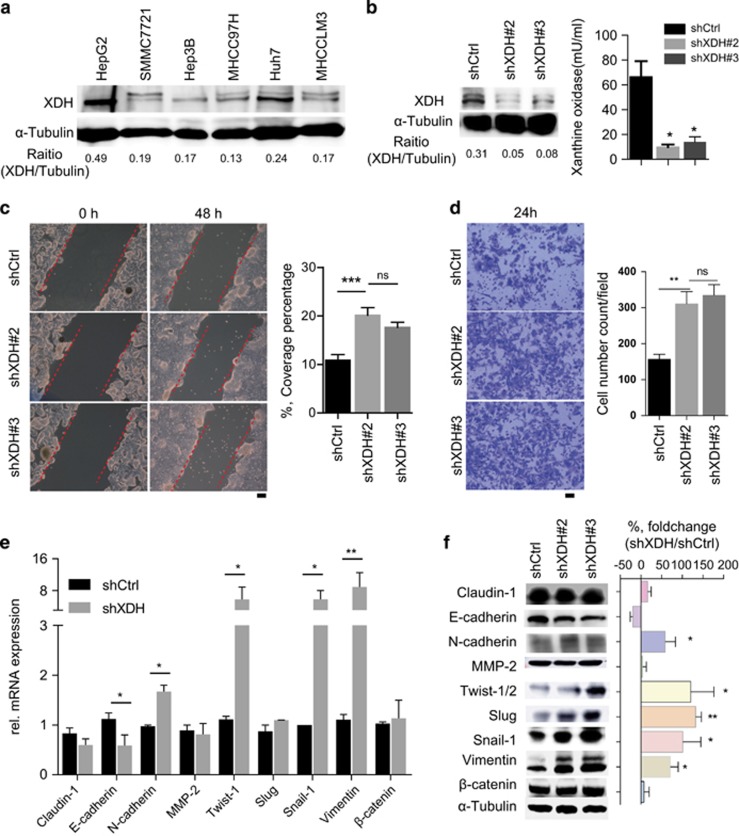
XDH knockdown increases cell migration and invasion in HepG2 cells. (**a**) Western blot analysis of XDH expression in HCC cell lines. The band intensities were quantified by ImageJ software. (**b**) Western blot analysis of XDH knockdown efficiency and quantification of XDH activity in HepG2 cells after shRNA transfection. (**c**, **d**) Scratch assay (**c**) and transwell assay (**d**) of the migration and invasion of HepG2 cells transfected with control shRNA (shCtrl) or shRNA against XDH (shXDH). Coverage percentages were determined, and quantitative analyses of the numbers of invading cells in each group were performed. Scale bar, 100 μm. (**e**, **f**) mRNA profiling (**e**) and western blot analysis (**f**) of EMT marker gene expression levels in HepG2 cells transfected with shRNA. XDH, xanthine dehydrogenase; EMT, epithelial-to-mesenchymal transition; mRNA, messenger RNA; HCC, hepatocellular carcinoma; shRNA, small-hairpin RNA; rel., relative. All data are expressed as the mean±s.e.m. of three experiments. Unpaired *t*-tests were performed to assess statistical significance. ns, not significant, *P<0.05, **P<0.01, ***P<0.001, ****P<0.0001.

To validate the findings of the database analysis, we first profiled XDH expression in HCC cell lines with different metastatic capacities. The HCC cell lines with a low metastatic capacity, such as the HepG2 and Huh7 cell lines, showed higher XDH expression than that of the cell lines with a high metastatic capacity, such as the MHCC97H and MHCCLM3 cell lines (Figure 2a). We subsequently knocked down XDH expression in HepG2 cells (Figure 2b). As shown in Figures 2c and d, knocking down XDH increased cell mobility and invasion in HepG2 cells but did not affect proliferation in these cells (Supplementary Figure 1a). To confirm these results at the molecular level, we performed mRNA profiling of a panel of EMT marker genes. This analysis revealed that the expression levels of epithelial marker genes, such as *E-cadherin*, were downregulated, whereas those of mesenchymal marker genes, such as *N-cadherin*, *Twist-1*, *snail-1* and *vimentin*, were significantly upregulated in HepG2 cells with stable XDH knockdown compared with those of control cells (Figure 2e). Western blot analysis of EMT marker expression also confirmed that E-cadherin expression levels were decreased, whereas N-cadherin, Twist-1/2, slug, snail-1 and vimentin expression levels were increased in HepG2 cells with stable XDH knockdown compared with those of the control cells (Figure 2f). In addition, oxypurinol reduced XDH activity (Supplementary Figure 1b) and promoted cell mobility and invasion but not proliferation in HepG2 cells (Supplementary Figure 1c–e). Similarity, oxypurinol-induced XDH inhibition promoted cell mobility and invasion but not proliferation in Huh7 cells (Supplementary Figure 2a–c). Consistent with these observations, quantitative real-time polymerase chain reaction (qRT–PCR) and western blot analysis of EMT marker genes also confirmed that EMT marker gene levels were increased in HepG2 (Supplementary Figure 1f, g) and Huh7 cells (Supplementary Figure 2d, e). Collectively, these data indicate that decreases in XDH expression or activity promote HCC cell invasiveness.

### XDH downregulation induces the TGFβ signaling pathway in HCC cells

**Figure 3:**
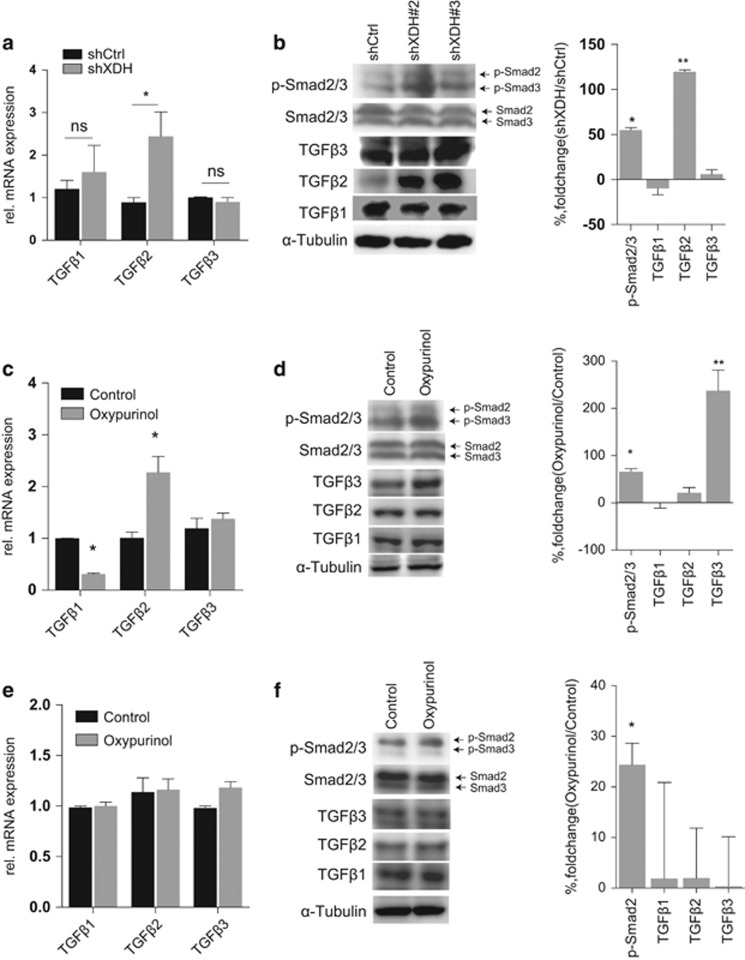
XDH negatively regulates the TGFβ-Smad signaling pathway in HCC cells. (**a**) qRT–PCR analysis of TGFβ isoform transcript expression in HepG2 cells. (**b**) Western blot analysis of TGFβ isoform and Smad2/3 phosphorylation levels in HepG2 cells transfected with control shRNA (shCtrl) or shRNA against XDH (shXDH). (**c**) qRT–PCR analysis of TGFβ isoform transcript expression in HepG2 cells in the absence or presence of 50 μM oxypurinol. (**d**) Western blot analysis of TGFβ isoform transcript and Smad2/3 phosphorylation levels in HepG2 cells in the absence or presence of 50 μM oxypurinol. (**e**) qRT–PCR analysis of TGFβ isoform transcript expression in Huh7 cells in the absence or presence of 50 μM oxypurinol. (**f**) Western blot analysis of TGFβ isoform transcript and Smad2/3 phosphorylation levels in Huh7 cells in the absence or presence of 50 μM oxypurinol. Quantitation of protein levels was performed using ImageJ software. All data are expressed as the mean±s.e.m. of three experiments. Unpaired *t*-tests were performed to assess statistical significance. XDH, xanthine dehydrogenase; qRT–PCR, quantitative reverse transcription polymerase chain reaction; HCC, hepatocellular carcinoma; TGFβ, transforming growth factor beta; Smad, mothers against decapentaplegic, drosophila; shRNA, small-hairpin RNA; rel., relative. ns, not significant, *P<0.05, **P<0.01, ***P<0.001, ****P<0.0001.

EMT marker gene upregulation is commonly associated with increases in TGFβ or β-catenin pathway activity in HCC.^40^ Correlation analysis of tumor samples in TCGA LIHC database showed that the *XDH* transcript levels were inversely correlated with the expression levels of molecules found in the TGFβ-Smads but not the β-catenin signaling pathway (Supplementary Table 4). To confirm these findings, we analyzed mRNA and protein levels in HCC cell lines. We found that neither knockdown nor inhibition of XDH resulted in significant changes in β-catenin mRNA and protein expression in HepG2 cells (Figures 2e and f, Supplementary Figure 1f, g) or Huh7 cells (Supplementary Figure 2d, e). TGFβ1 and TGFβ3 expression levels were comparable between XDH-specific small-hairpin RNA (shRNA)-transfected HepG2 cells and control cells, whereas TGFβ2 expression levels, as well as phosphorylated Smad2/3 levels, were increased in XDH-specific shRNA-transfected HepG2 cells compared with those in control cells (Figures 3a and b). In addition, oxypurinol treatment increased TGFβ2 mRNA levels, TGFβ3 protein levels and phosphorylated Smad2/3 protein levels but decreased TGFβ1 mRNA levels in HepG2 cells (Figures 3c and d). No changes in TGFβ mRNA and protein levels were observed in Huh7 cells treated with oxypurinol (Figures 3e and f). However, induction of phosphorylated Smad3 protein expression was observed in Huh7 cells after oxypurinol treatment (Figure 3f). Taken together, these data indicate that XDH deficiency may induce TGFβ signaling activation in HCC cells.

### Blocking TGFβ signaling abrogates XDH deficiency-induced cell migration and invasion in HCC cells

**Figure 4:**
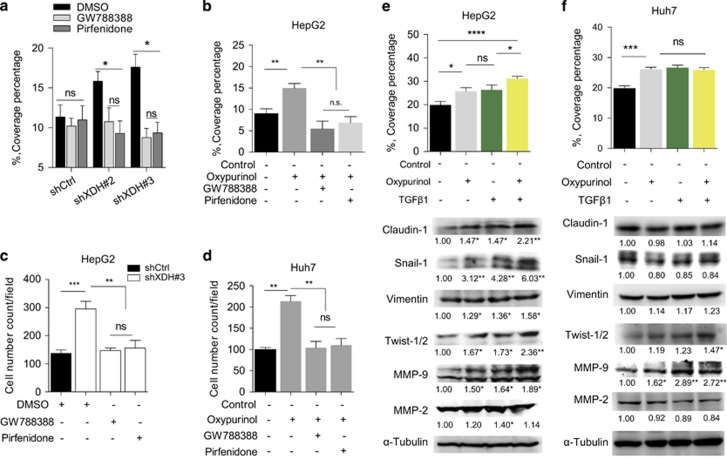
The effects of XDH downregulation on HCC cells are dependent on the TGFβ-Smad signaling pathway. (**a**) Scratch assay of the migration of HepG2 cells with stable XDH knockdown (shXDH) or control (shCtrl) cells treated with TGFβ pathway inhibitors (GW788388 or pirfenidone) for 48 h. (**b**) Scratch assay of the migration of HepG2 cells incubated in the presence or absence of 50 μM oxypurinol, 100 μM GW788388 or 2 nM pirfenidone. (**c**) Quantitative analysis of the numbers of invading cells in the XDH knockdown (shXDH) HepG2 and control (shCtrl) cell populations in the absence or presence of 100 μM GW788388 or 2 nM pirfenidone. (**d**) Quantitative analysis of the numbers of invading cells in the Huh7 cell population in the absence or presence of 100 μM GW788388, 2 nM pirfenidone or 50 μM oxypurinol. (**e**, **f**) Scratch assay of the migration of HepG2 cells (**e**) or Huh7 cells (**f**) treated with 50 μM oxypurinol, 5 ng/ml TGFβ1 or a combination of both for 48 h. Quantitative analyses of coverage percentages and western blot analyses and quantification of EMT marker expression levels in each group were performed. Unpaired *t*-tests were performed to assess statistical significance. All data are expressed as the mean±s.e.m. of three experiments. DMSO, dimethyl sulfoxide; EMT, epithelial-to mesenchymal transition; XDH, xanthine dehydrogenase; qRT–PCR, quantitative reverse transcription polymerase chain reaction; HCC, hepatocellular carcinoma; TGFβ, transforming growth factor beta; Smad, mothers against decapentaplegic, drosophila; shRNA, small-hairpin RNA. ns, not significant, *P<0.05, **P<0.01, ***P<0.001, ****P<0.0001.

Our observation of XDH downregulation-induced TGFβ signaling in HCC cells led us to ask whether this phenomenon is the key downstream effect of XDH knockdown or inhibition in HCC cells. As shown in Figure 4a, cell migratory ability, which was represented by coverage percentages, was comparable between HepG2 cells with stable XDH knockdown and control cells after GW788388 or pirfenidone was administered to block the TGFβ signaling pathway. Similarly, oxypurinol-induced increases in cell migration in HepG2 cells were abrogated by GW788388 or pirfenidone treatment (Figure 4b). Transwell invasion assays showed that the abovementioned increases in HepG2 cell migration were largely abrogated in the shXDH-transfected group compared with that of the control shRNA-transfected group after the addition of TGFβ signaling inhibitors (Figure 4c). TGFβ blockade also inhibited oxypurinol-induced cell invasion in Huh7 cells (Figure 4d). The effects of TGFβ1 alone or oxypurinol alone on HepG2 cell migration were comparable to those of control treatments (Figure 4e). However, treatment with a combination of TGFβ1 and oxypurinol resulted in high levels of HepG2 cell migration (Figure 4e), suggesting that oxypurinol and TGFβ1 exert synergistic effects on HepG2 cells. The combination of TGFβ1 and oxypurinol induced increases in cell migration that were comparable to those of TGFβ1 or oxypurinol alone in Huh7 cells (Figure 4f), suggesting that the cell migration-promoting effects of oxypurinol are dependent on the TGFβ signaling pathway. These observations were confirmed by our western blot analysis of changes in EMT marker gene expression in HepG2 cells (Figure 4e) and Huh7 cells (Figure 4f). These results suggest that XDH knockdown- or inhibition-induced cell migration and invasion are dependent on TGFβ-signaling pathway activation in HCC cells.

### XDH overexpression reduced TGFβ signaling, cell migration and invasion in MHCC97H cells *in vitro* and *in vivo*

**Figure 5:**
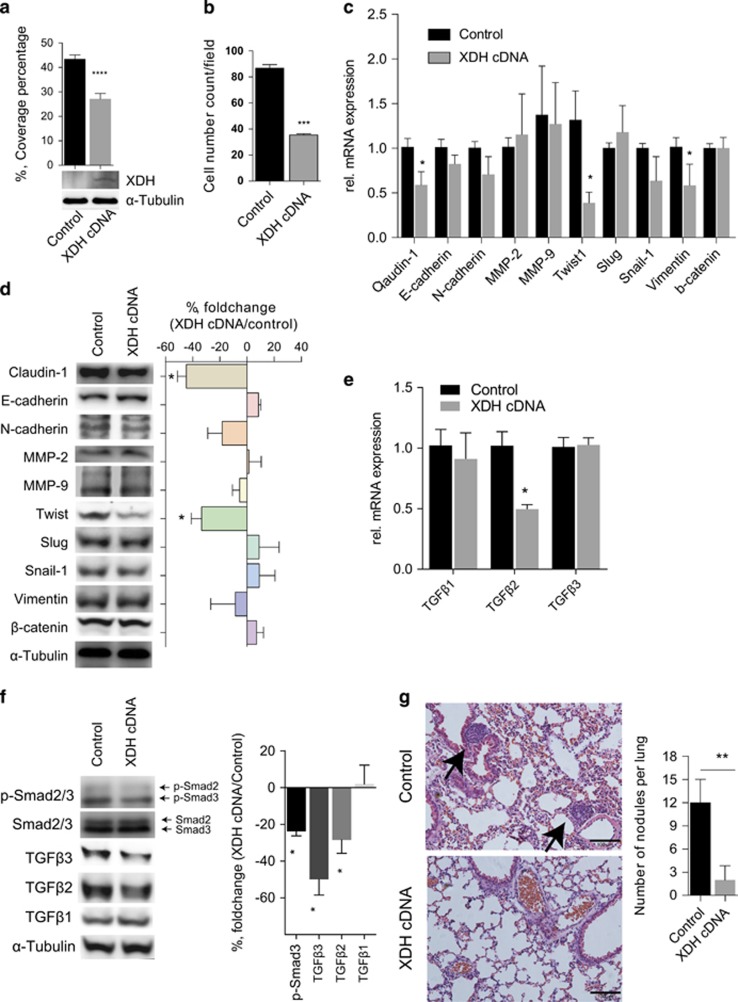
XDH overexpression decreases cell motility and invasion in MHCC97H cells. (**a**) XDH overexpression in MHCC97H cells was confirmed by western blot analysis. The decreases in cell migration were demonstrated by corresponding decreases in coverage percentages. (**b**) Quantitative analysis of the numbers of invading cells in MHCC97H cells with XDH overexpression or cells treated with control vectors. (**c**, **d**) Gene profiling (**c**) and western blot analysis (**d**) of EMT marker gene expression levels in MHCC97H cells transfected with cDNA. (**e**) qRT–PCR analysis of TGFβ isoform transcript expression in MHCC97H cells. rel., relative. (**f**) Western blot analysis of TGFβ isoform and Smad2/3 phosphorylation levels in MHCC97H cells. Quantitative analysis of protein expression levels was performed using ImageJ software. (**g**) Representative photograph of a hematoxylin–eosin-stained lung section in nude mice after tail vein injection of MHCC97H cells. Black arrows indicate the metastatic tumor foci in the lungs. Scale bar, 100 μm. *n*=8 for each group. Quantitative analyses of the numbers of nodules per lung were performed. XDH, xanthine dehydrogenase; EMT, epithelial-mesenchymal transition. Unpaired *t*-tests were performed to assess statistical significance. All *in vitro* data are expressed as the mean±s.e.m. of three experiments. XDH, xanthine dehydrogenase; EMT, epithelial-mesenchymal transition; qRT–PCR, quantitative reverse transcription polymerase chain reaction; HCC, hepatocellular carcinoma; TGFβ, transforming growth factor beta; Smad, mothers against decapentaplegic, drosophila. ns, not significant, *P<0.05, **P<0.01, ***P<0.001, ****P<0.0001.

To determine whether XDH upregulation downregulates cell mobility, invasion and TGFβ signaling in cell lines expressing XDH at low levels, we used a plasmid to overexpress XDH in MHCC97H cells. As shown in Figures 5a and b, XDH overexpression suppressed cell migration and invasion but not proliferation in MHCC97H cells compared with those of control cells (Supplementary Figure 3a). We noted that both the mRNA and protein expression levels of Claudin-1 were decreased in XDH-overexpressing MHCC97H cells compared with those of control cells (Figures 5c and d). Furthermore, we noted that *Twist-1* and *Vimentin* mRNA expression levels, as well as Twist protein expression levels, were decreased in XDH-overexpressing MHCC97H cells (Figures 5c and d), supporting the hypothesis that EMT marker gene expression can be blocked by XDH. XDH overexpression reduced TGFβ2 and TGFβ3 expression levels and phosphorylated Smad2 expression levels in MHCC97H cells (Figures 5e and f), indicating that XDH inhibits TGFβ signaling in HCC cells. Importantly, XDH-overexpressing MHCC97H cells resulted in a reduced numbers of metastatic nodules in the lungs (Figure 5g) but did not affect subcutaneous tumor growth (Supplementary Figure 3b), indicating that XDH may act as a tumor metastasis suppressor gene in HCC.

### Decreased XDH expression is associated with increased CSC-related gene expression

**Figure 6:**
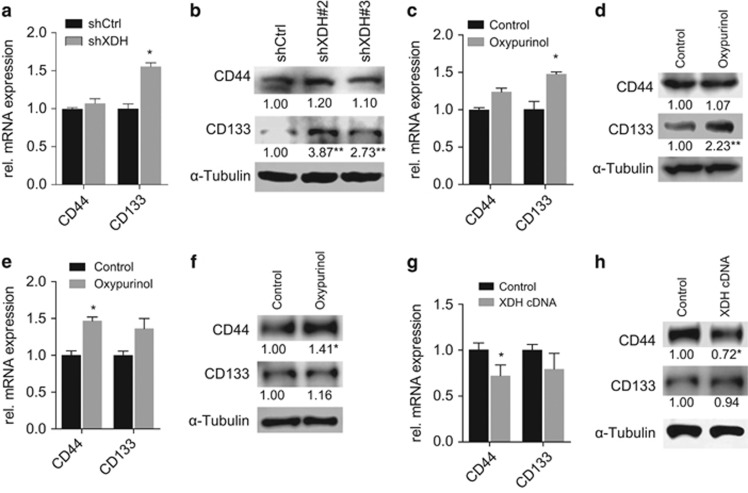
XDH regulates CSC marker gene expression levels in HCC cells. (**a**, **b**) qRT–PCR analysis of *CD44* and *CD133* mRNA expression levels (**a**) and western blot analysis of CD44 and CD133 protein expression levels (**b**) in HepG2 cells transfected with control shRNAs (shCtrl) or shRNAs against XDH (shXDH). rel., relative. (**c**, **d**) qRT–PCR analysis of *CD44* and *CD133* mRNA expression levels (**c**) and western blot analysis of CD44 and CD133 protein expression levels (**d**) in HepG2 cells in the presence of 50 μM oxypurinol for 48 h. (**e**, **f**) qRT–PCR analysis of *CD44* and *CD133* mRNA expression levels (**e**) and western blot analysis of CD44 and CD133 protein expression levels (**f**) in Huh7 cells treated with 50 μM oxypurinol or solvent (vehicle) only for 48 h. (**g**, **h**) qRT–PCR analysis of *CD44* and *CD133* mRNA expression levels (**g**) and western blot analysis of CD44 and CD133 protein levels (**h**) in MHCC97H cells transfected with control and XDH-overexpressing plasmids. The band intensities were quantified using ImageJ software. Unpaired *t*-tests were performed to assess statistical significance. All data are expressed as the mean±s.e.m. of three experiments. XDH, xanthine dehydrogenase; CSC, cancer stem cell; mRNA, messenger RNA; shRNA, small-hairpin RNA; qRT–PCR, quantitative reverse transcription polymerase chain reaction. ns, not significant, *P<0.05.

Our observation of XDH downregulation-induced EMT marker expression level and TGFβ-signaling activity upregulation raised the question of whether decreases in XDH expression levels affect CSC-related gene expression levels in HCC. Correlation analysis of a panel of eight CSC-related genes was performed in patient tumor samples (*n*=373) from TCGA LIHC. Interestingly, the mRNA expression levels of *XDH* were inversely correlated with the expression levels of all the genes in question (Supplementary Table 5). Upregulated CD133 mRNA levels and protein expression levels were consistently observed in HepG2 cells subjected to shRNA transfection or oxypurinol treatment compared with those of control cells (Figures 6a and d). Similarly, increased CD44 mRNA and protein expression levels were consistently observed in Huh7 cells treated with oxypurinol compared with those of control cells (Figures 6e and f). In contrast, decreased CD44 mRNA and protein expression levels were consistently observed in MHCC97H cells in which XDH was overexpressed (Figures 6g and h). Collectively, these data support the idea that XDH downregulation may be a critical molecular event in HCC development.

## Discussion

The impact of decreased XDH activity levels^41^ on the progression of HCC is poorly characterized. In this study, we showed that decreased XDH expression or activity could promote TGFβ-signaling pathway-dependent liver cancer cell migration, invasion and metastases to the lungs. In addition, decreased XDH expression is associated with increased CSC-related gene expression in HCC. These results may further elucidate how XDH downregulation promotes disease progression in HCC.

In the current study, we observed that decreased XDH expression or activity predisposes HCC to display an invasive phenotype, which is dependent on TGFβ-signaling activation. XDH loss in breast cancer cells increased the migratory ability of cancer cells, which is dependent on COX-2 and MMPs expression.^25^ Indeed, the TGFβ signaling pathway can induce COX-2 and MMP expression in liver tissue.^35, 42, 43, 44^ Moreover, we observed additive effects of XDH inhibition and TGFβ1 in HepG2 cells, which supports the increased response to TGFβ1 treatment in XDH-deficient breast cancer cells.^25^ Consistent with TGFβ signaling promotion of pulmonary metastasis of HCC,^45^ our data identified an important role of TGFβ-Smad2/3 signaling regulated by XDH in the process of HCC metastasis. However, how decreases in XDH expression occur during cancer progression and promote TGFβ signaling in HCC require future investigation. Whether XDH-derived oxidative stress or the antioxidant agent uric acid^46^ regulates the process of HCC metastasis remains unknown.

Here, our data and others^8^ suggested that loss of XDH expression contributes to cancer development and progression. In this regard, medications inhibiting XDH activity to reduce uric acid levels should be prescribed with caution for cancer patients or patients at risk for cancer in clinical settings. Although patients with non-alcoholic fatty liver disease^47^ or tumor lysis syndrome, as well as patients receiving cancer chemotherapy, will benefit from the use of XDH inhibitors because of the effects of these drugs on uric acid levels, long-term use of these drugs use may cause serious side effects in such patients.^8^ In support of this hypothesis, a recent retrospective cohort study found that use of allopurinol, an XDH/XOR inhibitor, for >3 months may significantly increase the incidence of both bladder cancer and all other cancers.^48^ Moreover, XDH inhibition by allopurinol may help cancer cells to escape immune surveillance.^49^ Although recent publications indicated that advanced cancer patients will benefit from XDH inhibition,^50, 51^ long-term follow-up of patients receiving XDH inhibitors treatment may be necessary to prevent detrimental outcomes.

However, the lack of studies examining the consequences of conditional XDH knockouts in hepatocytes^8^ *in vivo* prompted us to establish a causal relationship between XDH loss and the development and progression of HCC. We showed that XDH deficiency is a useful clinical indicator in patients with HCC and that XDH downregulation leads to TGFβ signaling pathway activation. Our findings may also be important for analysis of the pathogenesis of other cancers with decreased XDH expression levels.^8^ Future research on how XDH regulates TGFβ signaling might lead to new therapeutic targets for HCC.

## Materials and methods

### Cell lines

The indicated human HCC cell lines (HepG2, Hep3B, Huh7, SMMC-7721, MHCC97H and MHCCLM3) were maintained in either Dulbecco’s modified Eagle’s medium (Gibco, Shanghai, China, cat. no. 11965092) or minimum essential medium (Gibco, cat. no. 32561037) supplemented with 10% fetal bovine serum (Gibco, cat. no. 10270106), 100 units/ml penicillin and 100 μg/ml streptomycin at 37 °C with 5% CO_2_ in an incubator. The HepG2 and Hep3B cell lines were purchased from the Cell Resource Center, Institute of Biochemistry and Cell Biology, Chinese Academy of Sciences, Shanghai, China, whereas the MHCC97H, MHCCLM3, SMMC-7721 and Huh7 cell lines were generously donated by the Liver Cancer Institute of Fudan University, Zhongshan Hospital, Shanghai, China. The identity of the cell lines was authenticated with short tandem repeats profiling (FBI, CODIS). There were no signs of mycoplasma contamination in all cell lines.

### Cell proliferation

A WST-1 Cell Proliferation and Cytotoxicity Assay Kit (Beyotime Institute of Biotechnology, China) was used to detect HCC cell proliferation, as described in our previous report.^52^ In brief, the abovementioned cells were seeded in 96-well culture plates at a density of 2000 cells/well. To evaluate the effects of oxypurinol (Sigma-Aldrich, Co. LLC., Shanghai, China, cat. no. O6881), a potent xanthine oxidase inhibitor,^53^ on cell proliferation, we incubated the cells with or without 50 μmol/l (μM) oxypurinol. Cell proliferation was monitored over a 72-h time period and measured according to the manufacturer’s instruction. All experiments were performed at least three times and in triplicate.

### Cell transfection

MHCC97H cells were transfected with an EX-Mm05336-Lv201 plasmid (GeneCopoeia, Inc., Guangzhou, China) encapsulated in Lipofectamine 3000 Reagent (Invitrogen, Shanghai, China, cat. no. L3000015), according to a corresponding transfection protocol, to induce XDH overexpression or a pEZ-Lv201 control vector, which served as a negative control. Similarly, HepG2 cells were transfected with shRNA against XDH to knockdown XDH expression (shXDH) or control shRNA in a lentiviral vector (Biogot Technology, Co., Ltd., Nanjing, China). Stably transfected cells were selected using 1–2 μg/ml puromycin (InvivoGen, Shanghai, China, cat. no. ant-pr-1) for 2 weeks.

### Cell migration and invasion assays

For scratch assays, the cells were seeded in six-well plates with regular media. Serum-free media were used to avoid the confounding effects of proliferation on the results of the assay. A single scratch was made on the cell surface within each well using the tip of a sterile 200-μl pipette tip, after which the cells were washed with phosphate-buffered saline and cultured in regular media with 10% fetal bovine serum in the presence or absence of 50 μM oxypurinol, 2 mM GW788388 (Selleck Chemicals, Shanghai, China, cat. no. S2750), 100 nM pirfenidone (Selleck Chemicals, cat. no. S2907) or 5 ng/ml recombinant human TGFβ1 (PeproTech, Rocky Hill, NJ, USA, cat. no. 100-21C) for 48 h. Coverage percentages were determined by quantifying the open wound area percentages using CellSens microscope imaging software (Olympus Imaging America Inc., Center Valley, PA, USA). For transwell invasion assays, the cells (3.0–5.0 × 10^5^ per well) were suspended in medium without serum and seeded on 8-μm membrane inserts pre-coated with basement membrane extract (Trevigen, Gaithersburg, MD, USA, cat. no. 3455-096-02). The inserts were placed in wells with complete Dulbecco’s modified Eagle’s medium containing 10% fetal bovine serum, which served as a chemoattractant. After 24–48 h, the inserts were removed, washed with phosphate-buffered saline, fixed in methanol and then stained with crystal violet (0.05% w/v in methanol). The bottom surfaces of the stained inserts were subsequently observed under a light microscope, and the numbers of stained cells were counted in five fields/insert.

### Mouse experiment

Male BALBc/nu mice (5 weeks old) were purchased from SLAC (Shanghai Laboratory Animal Co., Ltd., Shanghai, China) and maintained in a specific pathogen-free environment at 25 °C under a 12-h light/dark cycle. No statistical methods were used to estimate sample size. The procedures used for the intravenous and subcutaneous injections were described previously by Zhang Y *et al.*^54^ and Wang F *et al.*,^55^ respectively. In brief, the nude mice were injected with 8 × 10^5^ MHCC97H cells stably overexpressing XDH or control vectors in 0.2 ml of phosphate-buffered saline via the lateral tail vein (*n* = 8 per group). Mice were randomized into the control vector group or XDH overexpression group. After 5 weeks, all the mice were sacrificed. Their lung tissues were dissected and fixed in 10% formalin for at least 24 h. The number of tumor colonies in each hematoxylin and eosin-stained lung tissue specimen was determined using a dissecting microscope. To evaluate *in vivo* tumor growth, we subcutaneously injected 8 × 10^6^ MHCC97H cells transfected with XDH-overexpression plasmids or control vectors in 0.2 ml of phosphate-buffered saline into the left or right flanks of nude mice (*n* = 8 per group). These mice were sacrificed at 3 weeks post injection. The volumes of the subcutaneous tumors were blindly measured and calculated using the equation length × width × width/2. All animal experiments were approved by the Animal Care and Use Committee of Minhang Hospital, Fudan University, Shanghai.

### Xanthine oxidase detection assay

A Xanthine Oxidase Assay Kit (ScienCell, Carlsbad, CA, USA, cat. No. 8458) was used to detect XDH activity in HCC cells after oxypurinol treatment or stable XDH knockdown. In brief, the HCC cells (4 × 10^6^ cells) were homogenized on ice using a Dounce homogenizer and treated with four volumes of assay buffer. The cells were subsequently centrifuged, and the supernatant was collected for xanthine oxidase activity measurements, according to the manufacturer’s instructions.

### qRT–PC

mRNA isolation and quantification were performed as previously reported.^52, 56^ The samples were analyzed in triplicate. β-actin and 18 S RNA were used as housekeeping genes. The primer sequences are listed in Supplementary Table 1.

### Western blotting

Cell lysates were prepared as previously reported.^56^ For western blotting, whole-cell lysates (20–40 μg per well) were separated by 10% sodium dodecyl sulfate–polyacrylamide gel electrophoresis. The resolved proteins were transferred to 0.2-μm polyvinylidene difluoride membranes, which were subsequently immersed in Quickblock blocking buffer (Beyotime Biotechnology, China, cat. no. P0233) for 0.5–1 h at room temperature. The membranes were then incubated with the appropriate primary antibodies (Supplementary Table 2) overnight at 4 °C before being incubated with the appropriate horseradish peroxidase-labeled secondary antibodies (Beyotime Biotechnology) for 2 h at room temperature. The bands were detected using a BeyoECL Plus Chemiluminescence Detection Kit (Beyotime Biotechnology). Images were acquired using an Amersham Imager 600 (GE Healthcare, Russellville, AR, USA).

### Immunohistochemical analysis

The immunohistochemical staining procedure was performed as previously described.^52, 56^ HCC liver samples were obtained after patients provided written informed consent, according to a protocol approved by the ethics committee of Zhongshan Hospital, Fudan University. The pathological tissue sections were stained with antibodies against XDH (Santa Cruz Biotechnology, Dallas, TX, USA, Cat. # sc-398548) at a 1:200 dilution. Images were acquired using a Nikon Eclipse 80i microscope equipped with a Sony DXC-390 P digital camera and NIS-Elements BR2.2 software.

### Public database analysis

Liver cancer gene expression data (mRNA, RNAseq *z*-scores) were retrieved from liver HCC data sets (LIHC) (Provisional) in the TCGA database using the UCSC Cancer Genomics Browser^54^ or the cBioPortal for Cancer Genomics.^57, 58^ Data pertaining to XDH expression abundance, which was found in the Gene Expression Omnibus data sets (GSE6764),^38^ were downloaded from the web-accessible Gene Expression across Normal and Tumor tissue (GENT) database.^59^ The EMT score was calculated by determining the difference between the expression levels of well-known mesenchymal marker genes and the total expression levels of known epithelial genes.^39^

### Statistical analysis

All results are presented as the mean±s.e.m. Statistical significance was determined using unpaired Student’s *t-*tests, the Mann–Whitney *U-*test, or one-way analysis of variance with Sidak’s or Tukey’s post-test (two-tailed). All graphs were generated, and all statistical analyses were performed using Prism software (GraphPad Software, Inc. La Jolla, CA, USA). For all analyses, *P*<0.05 was considered statistically significant (*P<0.05, **P<0.01, ***P<0.001, ****P<0.0001).
