# Supplementary material for: Xanthine dehydrogenase downregulation promotes TGFβ signaling and cancer stem cell-related gene expression in hepatocellular carcinoma
Source: Oncogenesis. 2017 Sep 25;6(9):e382–. doi: 10.1038/oncsis.2017.81 (PMC5623907; doi:10.1038/oncsis.2017.81)
Supplement: Supplementary Figure Legends [file oncsis201781x1.docx]

**Supplementary Figure Legends**

**Supplementary Figure 1 XDH inhibition induced cell migration and invasion in HepG2 cells.** (a) Proliferation assay of HepG2 cells transfected with shRNA (shXDH). (b) Analysis of XDH activity in HepG2 cells in the presence or absence of 50 μM oxypurinol. (c) Proliferation assay of HepG2 cells in the presence or absence of 50 μM oxypurinol. (d-e) Wound healing assay (d) or transwell assay (e) of cell migration or invasion in HepG2 cells in the presence of 50 μM oxypurinol. (f-g) mRNA profiling (f) and western blot analysis (g) of EMT marker gene expression levels in HepG2 cells in the presence of 50 μM oxypurinol. XDH, xanthine dehydrogenase; EMT, epithelial-mesenchymal transition; mRNA, messenger RNA; shRNA, small hairpin RNA; rel., relative. Unpaired *t*-tests were performed to assess statistical significance. All data are expressed as the mean ± s.e.m. of three experiments. ns, not significant, *p<0.05, **p<0.01.

**Supplementary Figure 2 XDH inhibition promotes cell migration and invasion in Huh7 cells.** (a) Proliferation assay of Huh7 cells in the presence of 50 μM oxypurinol. (b-c) Wound healing assay (b) or transwell assay (c) of cell migration or invasion in Huh7 cells in the presence of 50 μM oxypurinol. (d-e) mRNA profiling (d) and western blot analysis (e) of EMT marker gene expression levels in Huh7 cells treated with 50 μM oxypurinol. XDH, xanthine dehydrogenase; EMT, epithelial-mesenchymal transition; mRNA, messenger RNA; rel., relative. Unpaired *t*-tests were performed to assess statistical significance. All data are expressed as the mean ± s.e.m. of three experiments. ns, not significant, *p<0.05, **p<0.01.

**Supplementary Figure 3 XDH expression does not affect proliferation in MHCC97H cells *in vitro* or *in vivo*.** (a) Proliferation assay in MHCC97H cells with XDH overexpression or cells transfected with control vectors. (b) Tumor volumes in nude mice subcutaneously injected with MHCC97H cells on day 21. n=8 for each group. Unpaired *t*-tests were performed to assess statistical significance. XDH, xanthine dehydrogenase. ns, not significant.
