# Supplementary figures and images for: Xanthine dehydrogenase downregulation promotes TGFβ signaling and cancer stem cell-related gene expression in hepatocellular carcinoma
Source: Oncogenesis. 2017 Sep 25;6(9):e382–. doi: 10.1038/oncsis.2017.81 (PMC5623907; doi:10.1038/oncsis.2017.81)

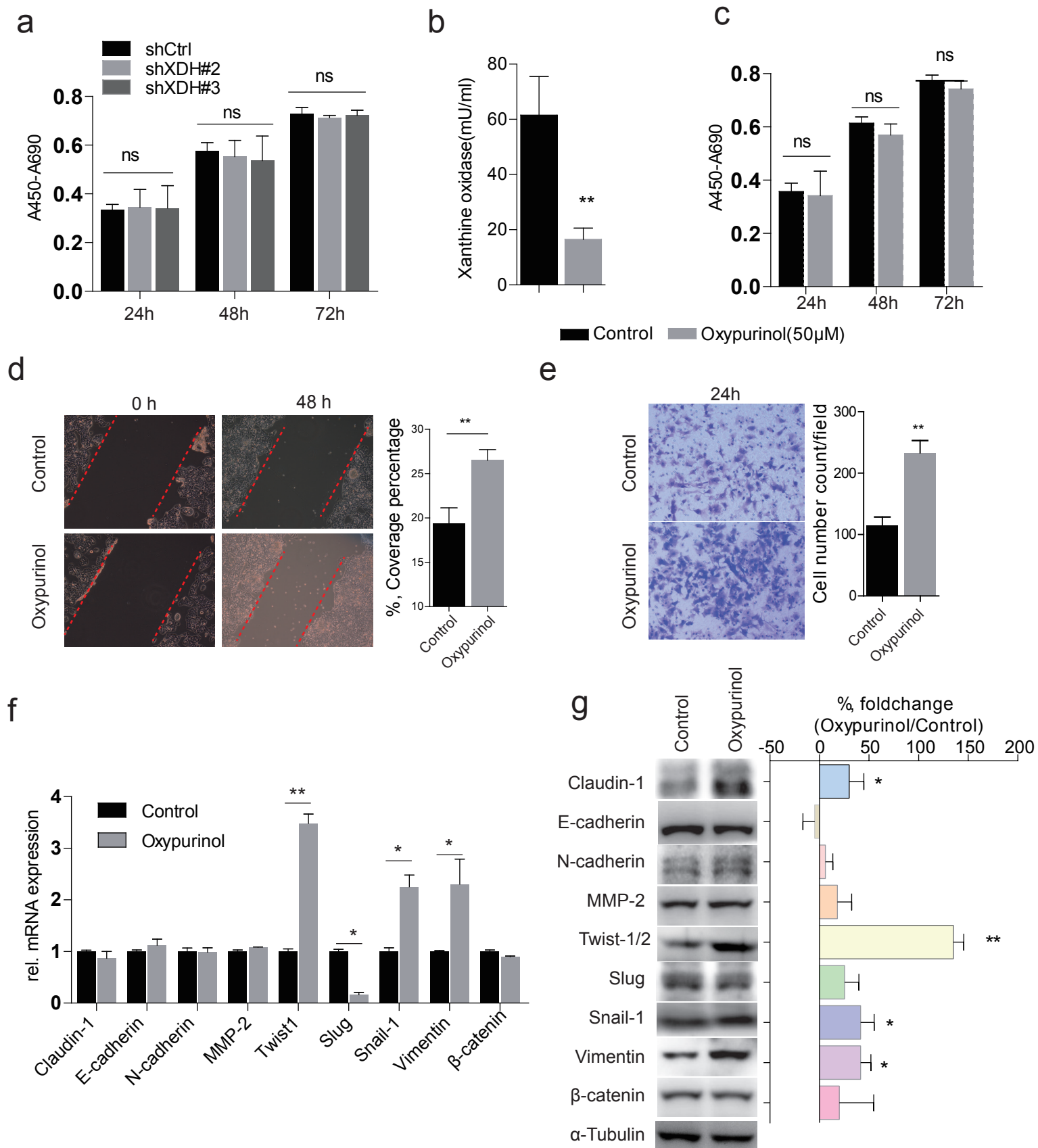

Supplement: Supplementary Figure 1 [file oncsis201781x2.pdf]

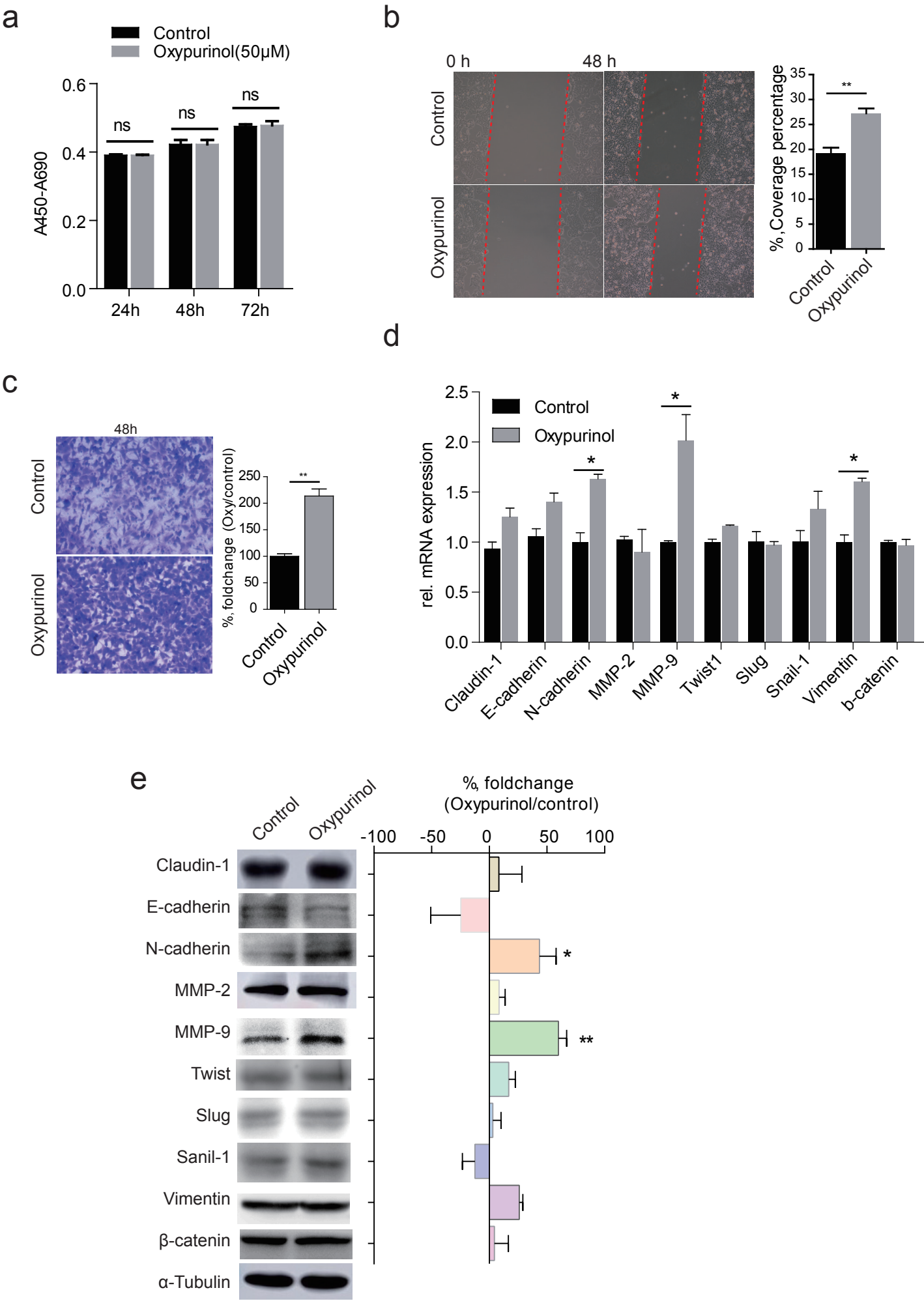

Supplement: Supplementary Figure 2 [file oncsis201781x3.pdf]

a

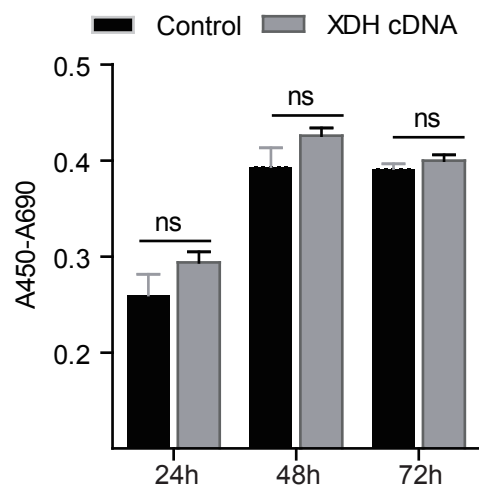

b

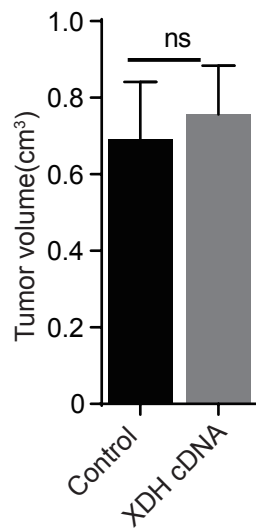

Supplement: Supplementary Figure 3 [file oncsis201781x4.pdf]
