# Supplementary material for: Xanthine dehydrogenase downregulation promotes TGFβ signaling and cancer stem cell-related gene expression in hepatocellular carcinoma
Source: Oncogenesis. 2017 Sep 25;6(9):e382–. doi: 10.1038/oncsis.2017.81 (PMC5623907; doi:10.1038/oncsis.2017.81)
Supplement: Supplementary Tables [file oncsis201781x5.docx]

**Supplementary Tables**

**Supplementary Table 1 List of the human primer sequences used for quantitative RT-PCR.**

| Gene | Forward (5' to 3') | Reverse (5' to 3') |
| --- | --- | --- |
| *Claudin-1* | GGAGACGACAAAGTGAAGAAGGC | AAAAGTCTGTGACAATCTGATGGC |
| *E-Cadherin* | GCCTCCTGAAAAGAGAGTGGAAG | TGGCAGTGTCTCTCCAAATCCG |
| *N-Cadherin* | CCTCCAGAGTTTACTGCCATGAC | GTAGGATCTCCGCCACTGATTC |
| *MMP-2* | AGCGAGTGGATGCCGCCTTTAA | CATTCCAGGCATCTGCGATGAG |
| *MMP-9* | GCCACTACTGTGCCTTTGAGTC | CCCTCAGAGAATCGCCAGTACT |
| *Twist* | GCCAGGTACATCGACTTCCTCT | TCCATCCTCCAGACCGAGAAGG |
| *XDH* | GGACAGTTGTGGCTCTTGAGGT | GGAAGGTTGGTTTTGCACAGCC |
| *Slug* | ATCTGCGGCAAGGCGTTTTCCA | GAGCCCTCAGATTTGACCTGTC |
| *Snail* | TGCCCTCAAGATGCACATCCGA | GGGACAGGAGAAGGGCTTCTC |
| *Vimentin* | AGGCAAAGCAGGAGTCCACTGA | ATCTGGCGTTCCAGGGACTCAT |
| *β-Catenin* | CACAAGCAGAGTGCTGAAGGTG | GATTCCTGAGAGTCCAAAGACAG |
| *18S rRNA* | GTAACCCGTTGAACCCCATT | CCATCCAATCGGTAGTAGCG |
| *β-actin* | CATTGCTGACAGGATGCAGAAGG | TGCTGGAAGGTGGACAGTGAGG |
| *TGFβ1* | TACCTGAACCCGTGTTGCTCTC | GTTGCTGAGGTATCGCCAGGAA |
| *TGFβ2* | AAGAAGCGTGCTTTGGATGCGG | ATGCTCCAGCACAGAAGTTGGC |
| *TGFβ3* | CTAAGCGGAATGAGCAGAGGATC | TCTCAACAGCCACTCACGCACA |
| *CD44* | CCAGAAGGAACAGTGGTTTGGC | ACTGTCCTCTGGGCTTGGTGTT |
| *CD133* | CACTACCAAGGACAAGGCGTTC | CAACGCCTCTTTGGTCTCCTTG |

**Supplementary Table 2 List of the primary antibodies and dilutions used in the study.**

| Antibody | Source | Catalogue # | Dilution |
| --- | --- | --- | --- |
| Claudin-1 | Cell Signaling Technology, Inc. | #4933 | 1:1000 |
| E-Cadherin | Cell Signaling Technology, Inc. | #3195 | 1:1000 |
| N-Cadherin | Cell Signaling Technology, Inc. | #13116 | 1:1000 |
| MMP-2 | Abcam | ab97779 | 1:1000 |
| MMP-9 | Abcam | ab137651 | 1:1000 |
| Twist | Santa Cruz Biotechnology | sc-15393 | 1:500 |
| Xanthine Oxidase | Santa Cruz Biotechnology | sc-20991 | 1:500 |
| Slug | Cell Signaling Technology, Inc. | #9585 | 1:1000 |
| Snail | Cell Signaling Technology, Inc. | #3879 | 1:1000 |
| Vimentin | Cell Signaling Technology, Inc. | #5741 | 1:1000 |
| β-Catenin | Cell Signaling Technology, Inc. | #8480 | 1:1000 |
| α-Tubulin | GeneTex Inc. | GTX102078 | 1:1000 |
| TGFβ1 | GeneTex Inc. | GTX110630 | 1:500 |
| TGFβ2 | GeneTex Inc. | GTX132546 | 1:500 |
| TGFβ3 | GeneTex Inc. | GTX111296 | 1:1000 |
| CD44 | GeneTex Inc. | GTX102111 | 1:1000 |
| CD133 | GeneTex Inc. | GTX100567 | 1:500 |
| Phospho-Smad2 (Ser465/467)  /Smad3 (Ser423/425) | Cell Signaling Technology, Inc. | #8828 | 1:1000 |
| Smad2/3 | Cell Signaling Technology, Inc. | #8685 | 1:1000 |

**Supplementary Table 3 XDH expression was inversely correlated with *matrix metalloproteinase (MMP)* gene expression levels in hepatocellular carcinoma livers from TCGA LIHC patient samples (n=373).**

| Gene name | Pearson r | P (two-tailed) | 95% confidence interval | Significant? |
| --- | --- | --- | --- | --- |
| *MMP-1* | -0.2929 | < 0.0001 | -0.3830 to -0.1972 | Yes |
| *MMP-2* | -0.1212 | 0.0192 | -0.2201 to -0.01992 | Yes |
| *MMP-3* | -0.1889 | 0.0002 | -0.2850 to -0.08908 | Yes |
| *MMP-7* | -0.1679 | 0.0011 | -0.2650 to -0.06753 | Yes |
| *MMP-9* | -0.2798 | < 0.0001 | -0.3709 to -0.1835 | Yes |
| *MMP-10* | -0.2072 | < 0.0001 | -0.3024 to -0.1079 | Yes |
| *MMP-11* | -0.3463 | < 0.0001 | -0.4326 to -0.2536 | Yes |
| *MMP-12* | -0.3212 | < 0.0001 | -0.4094 to -0.2270 | Yes |
| *MMP-14* | -0.198 | 0.0001 | -0.2936 to -0.09838 | Yes |
| *MMP-19* | -0.1484 | 0.0041 | -0.2462 to -0.04754 | Yes |
| *MMP-20* | -0.1185 | 0.0221 | -0.2175 to -0.01715 | Yes |
| *MMP-24* | -0.2139 | < 0.0001 | -0.3088 to -0.1149 | Yes |
| *TIMP1* | -0.09811 | 0.0584 | -0.1977 to 0.003494 | No |
| *TIMP2* | -0.1442 | 0.0053 | -0.2422 to -0.04329 | Yes |
| *TIMP3* | 0.1272 | 0.0139 | 0.02599 to 0.2259 | Yes |
| *TIMP4* | -0.1472 | 0.0044 | -0.2451 to -0.04632 | Yes |

**Supplementary Table 4 *XDH* mRNA levels were inversely correlated to molecules in the TGFβ-Smad signaling pathway in HCC livers in TCGA LIHC patient samples (n=373).**

| Gene name | Pearson r | P (two-tailed) | 95% confidence interval | Significant? |
| --- | --- | --- | --- | --- |
| *SMAD1* | 0.2589 | < 0.0001 | 0.1616 to 0.3512 | Yes |
| *SMAD2* | -0.1837 | 0.0004 | -0.2800 to -0.08367 | Yes |
| *SMAD3* | -0.2323 | < 0.0001 | -0.3262 to -0.1339 | Yes |
| *SMAD4* | 0.1755 | 0.0007 | 0.07523 to 0.2722 | Yes |
| *SMAD5* | 0.01791 | 0.7303 | -0.08381 to 0.1193 | No |
| *SMAD6* | -0.0807 | 0.1197 | -0.1808 to 0.02104 | No |
| *SMAD7* | -0.07197 | 0.1654 | -0.1723 to 0.02981 | No |
| *TGFα* | -0.09715 | 0.0609 | -0.1968 to 0.004465 | No |
| *TGFβ1* | -0.2457 | < 0.0001 | -0.3417 to -0.1510 | Yes |
| *TGFβ2* | -0.305 | < 0.0001 | -0.3943 to -0.2099 | Yes |
| *TGFβ3* | -0.1694 | 0.001 | -0.2664 to -0.06899 | Yes |
| *TGFβI* | -0.02523 | 0.6271 | -0.1265 to 0.07653 | No |
| *TGFβ1l1* | -0.1044 | 0.044 | -0.2038 to -0.002837 | Yes |
| *TGFβR1* | -0.0356 | 0.4931 | -0.1367 to 0.06621 | No |
| *TGFβR3* | 0.1922 | 0.0002 | 0.09249 to 0.2882 | Yes |
| *TGFβRAP1* | 0.07836 | 0.1309 | -0.02339 to 0.1785 | No |
| *POSTN* | -0.1447 | 0.0051 | -0.2427 to -0.04380 | Yes |
| *UBE2I* | -0.4849 | < 0.0001 | -0.5590 to -0.4032 | Yes |
| *TCF3* | -0.4905 | < 0.0001 | -0.5640 to -0.4093 | Yes |
| *CTNNB1* | -0.03813 | 0.4629 | -0.1392 to 0.06369 | No |
| *WNT10B* | -0.2898 | < 0.0001 | -0.3802 to -0.1940 | Yes |
| *KARS* | -0.369 | < 0.0001 | -0.4536 to -0.2778 | Yes |
| *ID2* | 0.4645 | < 0.0001 | 0.3809 to 0.5406 | Yes |
| *TAL1* | 0.1403 | 0.0066 | 0.03933 to 0.2385 | Yes |
| *EP300* | 0.1701 | 0.001 | 0.06974 to 0.2671 | Yes |

**Supplementary Table 5 XDH expression was inversely correlated with cancer stem cell (CSC) marker gene expression levels in hepatocellular carcinoma livers from TCGA LIHC patient samples (n=373).**

| Gene name | Pearson r | P (two-tailed) | 95% confidence interval | Significant? |
| --- | --- | --- | --- | --- |
| *AFP* | -0.2452 | < 0.0001 | -0.3383 to -0.1473 | Yes |
| *CD44* | -0.2359 | < 0.0001 | -0.3296 to -0.1376 | Yes |
| *CXCR4* | -0.2191 | < 0.0001 | -0.3137 to -0.1202 | Yes |
| *EpCAM* | -0.1991 | 0.0001 | -0.2947 to -0.09958 | Yes |
| *CD133* | -0.1971 | 0.0001 | -0.2928 to -0.09749 | Yes |
| *NF2* | -0.14 | 0.0067 | -0.2382 to -0.03903 | Yes |
| *Thy1* | -0.1028 | 0.0473 | -0.2022 to -0.001239 | Yes |
| *CD24* | -0.1124 | 0.03 | -0.2116 to -0.01097 | Yes |
